# Teflon bridge technique for endoscopic-assisted microvascular decompression of ectatic basilar artery and anterior inferior cerebellar artery for trigeminal neuralgia: operative video and technical nuances

**DOI:** 10.3171/2020.7.FOCVID2032

**Published:** 2020-10-01

**Authors:** James K. Liu, Asif Shafiq

**Affiliations:** Departments of 1 Neurological Surgery and; 2 Otolaryngology–Head and Neck Surgery,; 3 Center for Skull Base and Pituitary Surgery, Neurological Institute of New Jersey, Rutgers University, New Jersey Medical School, Newark; Saint Barnabas Medical Center, RWJ Barnabas Health, Livingston, New Jersey

**Keywords:** trigeminal neuralgia, microvascular decompression, endoscopic-assisted, ectatic basilar artery, operative video

## Abstract

In this illustrative operative video, the authors demonstrate a Teflon bridge technique to achieve safe transposition of a large, tortuous ectatic basilar artery (BA) and anterior inferior cerebellar artery (AICA) complex to decompress the root entry zone (REZ) of the trigeminal nerve in a 61-year-old woman with refractory trigeminal neuralgia via an endoscopic-assisted retractorless microvascular decompression. Postoperatively, the patient experienced immediate facial pain relief without requiring further medications. The Teflon bridge technique can be a safe alternative to sling techniques when working in narrow surgical corridors between delicate nerves and vessels. The operative technique and surgical nuances are demonstrated.

The video can be found here: https://youtu.be/hIHX7EvZc1c

## Transcript

This is Dr. James Liu, and I will be demonstrating the Teflon bridge technique for endoscopic-assisted microvascular decompression of an ectatic basilar artery and anterior inferior cerebellar artery for trigeminal neuralgia.

**0:35 Patient History and Physical Examination.** The patient is a 61-year-old female who presented with 12 years of left-sided type 1 trigeminal neuralgia in the V2/V3 region. She had some temporary relief with carbamazepine, but her pain became refractory to medical therapy. Her neurological exam was otherwise intact.

**0:55 Preoperative Imaging.** Preoperative FIESTA MRI demonstrated an ectatic basilar artery compressing the left trigeminal nerve at the root entry zone.^1^ The nerve appeared to be displaced superiorly toward the tentorium.

**1:12 Patient Positioning and Skin Incision.** The patient underwent a left retrosigmoid craniectomy for endoscopic-assisted microvascular decompression.^2–4^ A lateral park-bench position is used and a left-sided C-shaped skin incision is marked roughly three fingerbreadths behind the pinna. For this case, we used a larger skin incision to accommodate for a larger bone opening and dural opening, so that we would have more surgical degrees of freedom when working with a large ectatic basilar artery. Having a larger working corridor can also be useful in the event a vascular injury is encountered and needs to be addressed. Neuromonitoring was used with somatosensory evoked potentials, brainstem auditory evoked responses, and facial nerve EMG.

**1:53 Retrosigmoid Approach: Surgical Exposure.** A galeocutaneous flap is raised and the muscles are mobilized inferiorly to expose the subocciput. The asterion is a useful landmark for the transverse-sigmoid junction. A retrosigmoid craniectomy is performed to expose the posterior fossa dura and the edges of the transverse and sigmoid sinuses. The mastoid air cells are waxed off and the dural incision is made in a reverse C-shaped fashion.

**2:19 Microsurgical Intradural Exposure.** The dural leaflets are tacked up to open up the working corridor and the microscope is brought in for intradural dissection. CSF is released from the trigeminal cisterns for brain relaxation. This allows us to perform the microvascular decompression using a retractorless technique. The superior petrosal vein is identified in an extraarachnoid fashion and carefully coagulated and divided sharply as close to the tentorium as possible. This widens the corridor to allow us to open the arachnoid over the trigeminal nerve.

**3:07 Endoscopic View of the Neurovascular Conflict.** A 30° angled endoscope is used to inspect the anatomical relationships.

As predicted on the FIESTA MRI, a tortuous ectatic basilar artery is identified displacing the trigeminal nerve toward the tentorium.

**3:27 Microsurgical Exposure of the Ectatic Basilar Artery and Trigeminal Nerve.** We continue the arachnoid dissection of the trigeminal nerve with the microscope. The anterior inferior cerebellar artery is also compressing the trigeminal nerve more distally in the cisternal segment. We then begin dissecting the ectatic basilar artery away from the root entry zone of the trigeminal nerve. This is most likely the point of neurovascular conflict. It is clear that there is an indentation of the nerve caused by the basilar artery. Although we considered a Teflon sling to suspend the artery,^5^ the working corridor and orientation of the artery did not seem favorable for this technique without risk to the neighboring facial nerve.

**4:21 Teflon Bridge Technique to Decompress the Trigeminal Root.** We therefore used a Teflon bridge technique to decompress the trigeminal root. The idea is to build a bridge in a progressive manner to support the large pulsatile basilar artery so that it maintains its transposed position away from the root entry zone of the trigeminal nerve.

As we follow the course of the basilar artery, we continue to build the Teflon bridge along the course of the trigeminal nerve. This is important to ensure that there is no compression along the root entry zone. Using a Fukushima ball-tip dissector, we carefully position the Teflon sponges so that the Teflon bridge elevates the basilar artery away from the nerve without the sponges making direct contact with the nerve.

**5:24 Transposition of the Anterior Inferior Cerebellar Artery and Ectatic Basilar Artery With Teflon Bridge.** We now turn our attention to the anterior inferior cerebellar artery, which also appears to be a point of neurovascular conflict. This is mobilized inferiorly after opening up the arachnoid.

Additional Teflon sponges are interposed between the anterior inferior cerebellar artery and the point of compression along the trigeminal nerve. Lastly, another Teflon bridge is wedged between the loop of the basilar artery and the tentorium to maintain the arterial transposition away from the trigeminal root entry zone.

**6:18 Final Inspection of Decompression With Angled Endoscope.** Final inspection is performed with a 30° angled endoscope. We can confirm that the Teflon bridge construct has adequately transposed the basilar artery away from the trigeminal root entry zone. The seventh and eighth nerve complex as well as the lower cranial nerves at the jugular foramen are well visualized.

**6:51 Closure.** The microscope is brought back in, and a few drops of fibrin glue are placed over the Teflon. The dura is closed in a watertight fashion with a 4-0 Nurulon suture. The mastoid air cells are sealed with bone wax at the time of closure and an autologous fat graft is placed over the dural suture line and fills the craniectomy defect. This graft is then buttressed with a Medpor Titan cranioplasty to prevent CSF leakage.

**7:23 Postoperative Course.** Postoperative FIESTA MRI shows transposition of the ectatic basilar artery away from the tentorium and trigeminal root. Postoperatively, the patient was neurologically intact without CSF leakage. All trigeminal neuralgia medications were stopped immediately after surgery to assess facial pain outcome. She had no further trigeminal pain and remained pain free and off medications at 1 year after surgery.

**7:59 Conclusion.** In summary, the Teflon bridge technique with endoscopic assistance can be a useful strategy for retractorless microvascular decompression of a tortuous ectatic basilar artery for trigeminal neuralgia.

**8:13 References**

## Author Contributions

Primary surgeon: Liu. Editing and drafting the video and abstract: both authors. Critically revising the work: both authors. Reviewed submitted version of the work: both authors. Approved the final version of the work on behalf of both authors: Liu. Supervision: Liu.
